# Understanding patient non-transport decision theories in the pre-hospital setting: a narrative review

**DOI:** 10.1186/s12245-023-00528-7

**Published:** 2023-10-11

**Authors:** Hassan Farhat, Kawther El Aifa, Guillaume Alinier, Abdulqadir Nashwan, Padarath Gangaram, Moncef Khadhraoui, Loua Al-Shaikh, Imed Gargouri, James Laughton

**Affiliations:** 1 Hamad Medical Corporation Ambulance Service, Doha, Qatar; 2 Faculty of Sciences, University of Sfax, 3000 Sfax, Tunisia; 3 Faculty of Medicine of Sousse “Ibn El Jazzar”, University of Sousse, 4000 Sousse, Tunisia; 4 School of Health and Social Work, University of Hertfordshire, Hatfield, UK; 5 Weill Cornell Medicine-Qatar, Doha, Qatar; 6 Northumbria University, Newcastle Upon Tyne, UK; 7 Department of Nursing, Hamad Medical Corporation, Doha, Qatar; 8 Faculty of Health Sciences, Durban University of Technology, PO Box 1334, Durban, 4000 South Africa; 9 Higher Institute of Biotechnology, University of Sfax, Sfax, Tunisia; 10 Faculty of Medicine, University of Sfax, Sfax, Tunisia

**Keywords:** Non-conveyance, Non-transport, Transport refusal, Prehospital care, Ambulance service

## Abstract

**Background:**

In pre-hospital emergency care, decisions regarding patient non-conveyance emerged as significant determinants of healthcare outcomes and resource utilization. These complex decisions became integral to the progress of emergency medical services, thus warranting an evolving exploration within the medical discourse.

**Objectives and methods:**

This narrative review aimed to synthesize and critically evaluate various theoretical stances on patient non-conveyance in the pre-hospital emergency. The focus on studies published between January 2012 and August 2022 was intentional to capture contemporary practices and insights. PubMed and Google Scholar served as the primary databases for the investigation, while the AL-Rayyan® software facilitated a thorough screening process.

**Results and discussion:**

Twenty-nine studies—encompassing articles, books, and theses—were discovered through our search, each presenting unique perspectives on patient non-transport, thus highlighting its criticality as a healthcare concern. Predominant factors influencing non-transport decisions were classified into patient-initiated refusals (PIR), clinician-initiated decisions (CID), and dispatcher-initiated decisions (DID).

**Conclusions:**

The issue of patient non-conveyance to hospitals continues to pose a crucial challenge to the seamless operation of emergency healthcare systems, warranting increased attention from various healthcare entities. To comprehend and pinpoint potential areas of improvement, a comprehensive analysis of pre-hospital non-transport events is imperative. A well-informed, strategic approach could prevent resource waste while ensuring patients receive the required and definitive care.

**Key messages:**

**Why is this topic important?**

Some studies have suggested that non-transport to hospitals following emergency calls is safe. However, it is a concerning issue for health systems. It is also considered a key performance metric for health systems.

**What does this review attempt to show?**

This review aimed to map the various factors discussed in the literature regarding the decisions not to transport patients following emergency calls in a pre-hospital setting.

**What are the key findings?**

The existing theories regarding non-transport to hospitals after the provision of emergency care in the pre-hospital setting were identified. Non-transport due to non-clinical decisions jeopardizes emergency care outcomes for paediatric and elderly patients in particular. Hence, further research is required to identify and control the factors governing these decisions.

**How is patient care impacted?**

The decisions regarding patient transport following emergency calls in a pre-hospital setting are crucial for patient outcomes. They could impact the pre-hospital emergency care outcomes as well as patient safety. They can also affect the emergency services resources’ ability to respond to other critical emergencies.

## Background

The Emergency Medical Services (EMS) system provides an out-hospital emergency medical service response. When a health emergency arises, the patient or their next of kin will likely dial the EMS number. Most EMS services utilize a computerized dispatch system to manage pre-hospital emergency calls [1]. Once the emergency call is received, the emergency medical dispacther (EMD) processes the call using internationally recognized and standardized software systems [2]. Initially, the caller is asked a series of pre-determined questions using a Program Question Answer (ProQA®) system owned by Priority Dispatch Corporation.™ (PDC). These are used to determine the final dispatch coding [3]. Subsequently, the EMD determines the most appropriate emergency response unit (ERU) for immediate dispatch using the Medical Priority Dispatch Systems (MPDS). After ERU’s arrival on scene, the medical responder assesses and triages the patient, provides them with initial emergency treatment, and transports them to the appropriate healthcare facility to receive definitive care [4–6].

In most EMS systems, patients with full mental capacity have the absolute right to refuse treatment or transport to a healthcare facility. Recent studies have identified that the percentage of patients not transported to healthcare facilities varies between 3.7 and 93.7% globally [7]. The theory-based classification of non-transport factors could help explain the wide variance in non-transport decisions. Understanding these factors and theories is essential as non-transport concerns health systems worldwide [6–8].

Researchers have worked on determining appropriate guidance, regulations, and rules to mitigate the potential risks of non-transport [9, 10]. Such decisions could jeopardize patient health, delay definitive care, and create a feeling of fear within the EMS staff [4]. To our knowledge, no previous review summarized the existing theories of patient non-transportation following an emergency call in the pre-hospital setting.

This review highlights the different approaches published in the literature regarding patient non-transport, also called non-conveyance, to healthcare facilities in the pre-hospital emergency setting.

## Methods

A narrative review of the literature was conducted. First, a research question was formulated, followed by a pilot search to identify and map the theories reported in the literature. Subsequently, a thorough search was conducted between May and August 2022 through Google Scholar and MEDLINE/PubMed. Only articles published during the last decade (between 2012 and 2022) were included in this search. These inclusion criteria were adopted as, over the last decade, EMS systems have seen significant changes, including a surge in the use of new technologies, such as digital communications and electronic medical records, improvements in the health policies, medical guidelines and clinical practice [11, 12]. This could significantly impact decision-making processes regarding patient transport, and then including older articles may not reflect these realities. The PubMed search was conducted using MeSh terms (((non transport[Title] ambulance[Title]) OR (Non conveyance hospital[Title])) OR (non transport hospital[Title])) OR (Non conveyance[Title] hospital[Title]). The Google Scholar search was conducted using (allintitle: “Non conveyance “allintitle: “Non transport”). This helped to hone in on articles directly applicable to this review, as generally if non-transport or non-conveyance were a significant part of a study, it would be mentioned in the title. Articles where these topics were not in the title could be less focused on these aspects and not significantly contribute to our review.

All articles, books, theses, and reports discussing patients’ outcomes after non-transport were considered. However, studies that did not discuss patient non-transport outcomes in the pre-hospital setting were excluded. Al-Rayyan® software was also utilized in this study. Al-Rayyan® is a free web software that facilitates the screening of articles, titles, and abstracts using a semi-automatic process [13]. Articles pertinent to the subject were identified and imported into the Al-Rayyan® software. Subsequently, Al-Rayyan® automatically identified the duplicated articles and excluded them after verification. Afterwards, the authors (HF and KEA) reviewed and screened the remaining articles’ titles, abstracts, and full texts for relevance, with blinding initially turned off and then on in Al-Rayyan®. In case of disagreement, a third reviewer (GA) was consulted. The authors (HF and KEA performed the analysis as well. Articles with irrelevant backgrounds and outcomes were excluded, along with duplicates not identified by the Al-Rayyan® software.

## Results

**Fig. 1:**
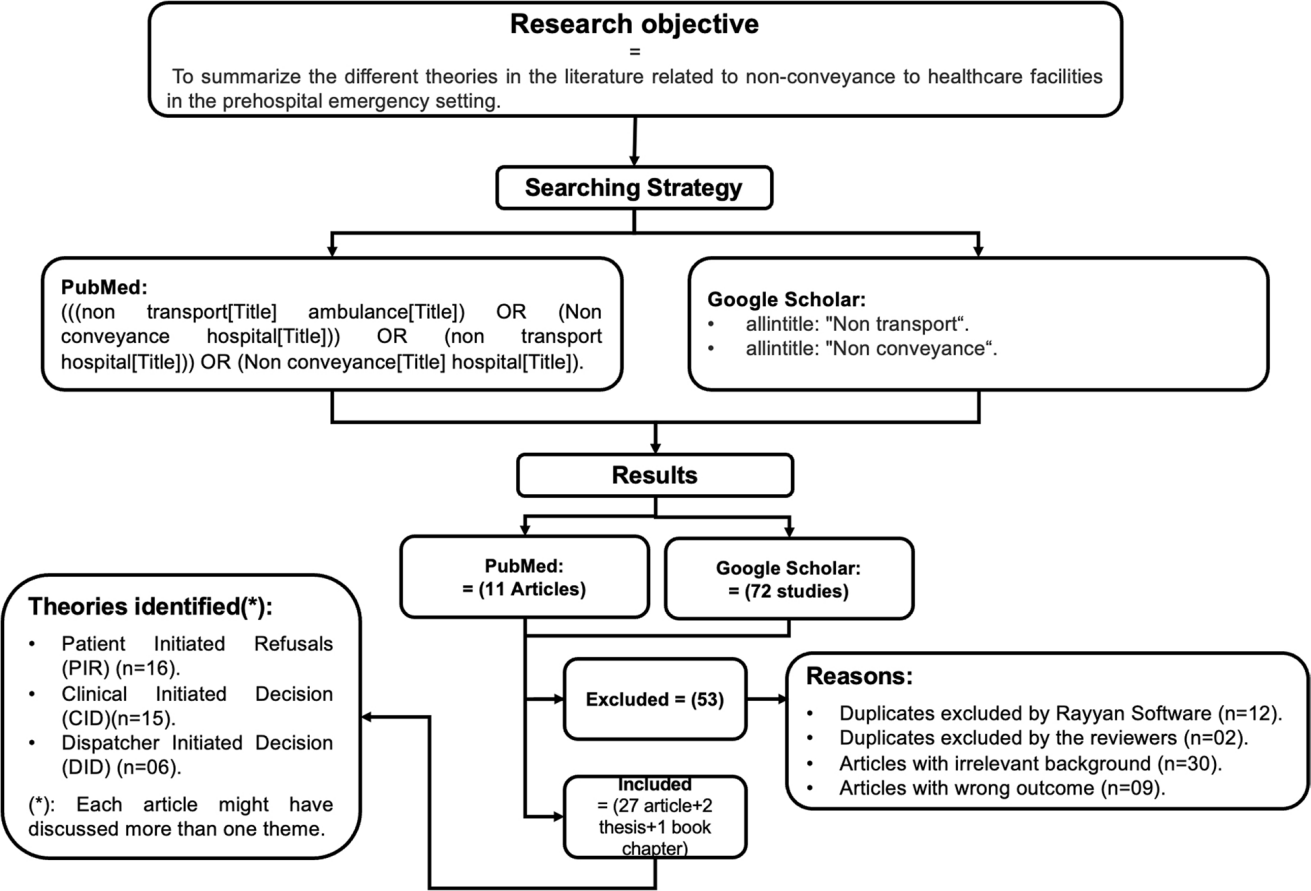
Search strategy

A total of 29 articles fulfilled the inclusion criteria. The retained articles and their identifiers are listed in Table 1 and Fig. 1.

**Table 1:** List of the articles included in this study

	Title	Reference type	Database provider	Year	Volume	Authors	ISBN/ISSN	DOI	URL
1	Can Paramedics Safely Refuse Transport of Non-Urgent Patients?	Journal Article	Cambridge University Press	2016	31	Fraess-Phillips, Alex J	1049-023X, 1945–1938	10.1017/S1049023X16000935	https://www.cambridge.org/core/journals/prehospital-and-disaster-medicine/article/abs/can-paramedics-safely-refuse-transport-of-nonurgent-patients/B0E2CA566A9B268DEA7A16BC0C6FF158#
2	Variation in Interpretation of Guidance as an Explanation of Between-Service Variation in Ambulance Quality Indicators on Non-Conveyance	Journal Article	emj.bmj.com	2016	33	Stone, Tony; O’Cathain, Alicia; Knowles, Emma	1472–0205, 1472–0213	10.1136/emermed-2016–206139.36	https://emj.bmj.com/content/33/9/e11.2;
3	A patient-safety and professional perspective on non-conveyance in ambulance care: a systematic review	Journal Article	BioMed Central	2017	25	Ebben, Remco H.A.; Vloet, Lilian C.M.; Speijers, Renate F.; Tönjes, N	1757–7241	10.1186/s13049-017–0409-6	https://doi.org/10.1186/s13049-017-0409-6;
4	Conveyance and non-conveyance to the emergency department after self-harm: Prevalence and ambulance service staff perspectives	Thesis	etheses.whiterose.ac.uk	2017	Unassigned	Jenkins, Emily Jayne	Unassigned	Unassigned	https://etheses.whiterose.ac.uk/18257/;
5	Encountering and counselling patients and family members in out-of-hospital emergency care in non-conveyance situations	Journal Article	pagepressjournals.org	2018	14	Paavilainen, Eija; Mikkola, Riitta; Salminen-Tuomaala, Mari; Leikkol	2282–2054	10.4081/ecj.2018.7468	https://pagepressjournals.org/index.php/ecj/article/view/7468;
6	Exploring variation in how ambulance services address non-conveyance: a qualitative interview study	Journal Article	bmjopen.bmj.com	2018	8	Knowles, Emma; Bishop-Edwards, Lindsey; O’Cathain, Alicia	2044–6055, 2044–6055	10.1136/bmjopen-2018–024228	https://bmjopen.bmj.com/content/8/11/e024228;
7	Implementation and use of computerized clinical decision support (CCDS) in emergency pre-hospital care: a qualitative study of paramedicine	Journal Article	BioMed Central	2018	13	Porter, Alison; Dale, Jeremy; Foster, Theresa; Logan, Pip; Wells, Bridg	1748–5908	10.1186/s13012-018–0786-x	https://doi.org/10.1186/s13012-018-0786-x;
8	Non-transport emergency medical service missions – a retrospective study based on medical charts	Journal Article	Wiley Online Library	2018	62	Pekanoja, S.; Hoikka, M.; Kyngäs, H.; Elo, S	1399–6576	10.1111/aas.13071	https://onlinelibrary.wiley.com/doi/abs/10.1111/aas.13071;
9	Variation in non-conveyance of patients with breathing problems (work package 4.2)	Book	www.ncbi.nlm.nih.gov	2018	Unassigned	O’Cathain, Alicia; Knowles, Emma; Bishop-Edwards, Lindsey; Coster,	Unassigned	Unassigned	https://www.ncbi.nlm.nih.gov/books/NBK506836/;
10	Why do ambulance services have different non-transport rates? A national cross-sectional study	Journal Article	PLoS Journals	2018	13	O’Cathain, Alicia; Jacques, Richard; Stone, Tony; Turner, Janette	1932–6203	10.1371/journal.pone.0204508	https://journals.plos.org/plosone/article?id=10.1371/journal.pone.0204508;
11	Characteristics of non-conveyance ambulance runs:A retrospective study in the Netherlands	Journal Article	PubMed Central	2019	10	Ebben, Remco H.A.; Castelijns, Mariola; Frenken, Joost; Vloet, Lilian	1920–8642	10.5847/wjem.j.1920–8642.2019.04.008	https://www.ncbi.nlm.nih.gov/pmc/articles/PMC6732166/;
12	Public attitudes towards the preventability of transport and non-transport related injuries: Can a social marketing campaign make a differnce	Journal Article	ScienceDirect	2019	13	Karbakhsh, Mojgan; Beaulieu, Emilie; Smith, Jennifer; Zheng, Alex; T	2211–3355	10.1016/j.pmedr.2018.12.010	https://www.sciencedirect.com/science/article/pii/S2211335518,301,591;
13	Patients’ aged ≥ 65 years dispositions during ambulance assignments, including factors associated with non-conveyance to hospital	Journal Article	BMJ	2020	10	Forsgärde, Elin-Sofie; Elmqvist, Carina; Fridlund, Bengt; Svensson, A	2044–6055, 2044–6055	10.1136/bmjopen-2020–038885	https://bmjopen.bmj.com/content/10/11/e038885;
14	Association Between an Electronic Non-transport Checklist and the Mortality of Patients Discharged-at-Scene by Paramedics in New Zealand	Thesis	Auckland University of Techn	2021	Unassigned	Watson, Fraser	Unassigned	Unassigned	https://openrepository.aut.ac.nz/handle/10292/14360;
15	EMS non-conveyance: A safe practice to decrease ED crowding or a threat to patient safety?	Journal Article	Springer	2021	21	Paulin, Jani; Kurola, Jouni; Koivisto, Mari; Iirola, Timo	1471-227X	10.1186/s12873-021–00508-1	https://doi.org/10.1186/s12873-021-00508-1;
16	Non-Conveyance Due to Patient-Initiated Refusal in Emergency Medical Services: A Retrospective Population-Based Registry Analysis	Journal Article	MDPI	2021	18	Moafa, Hassan N.; van Kuijk, Sander M. J.; Moukhyer, Mohammed E	1660–4601	10.3390/ijerph18179252	https://www.mdpi.com/1660-4601/18/17/9252;
17	Non-conveyance of older adult patients and association with subsequent clinical and adverse events after initial assessment by ambulance	Journal Article	BioMed Central	2021	21	Lederman, Jakob; Lindström, Veronica; Elmqvist, Carina; Löfvenmark	1471-227X	10.1186/s12873-021–00548-7	https://doi.org/10.1186/s12873-021-00548-7;
18	Patient experience of non-conveyance following emergency ambulance service response: A scoping review of the literature	Journal Article	ScienceDirect	2021	24	King, Robbie; Oprescu, Florin; Lord, Bill; Flanagan, Belinda	2588-994X	10.1016/j.auec.2020.08.006	https://www.sciencedirect.com/science/article/pii/S2588994X2030083X;
19	The Alternative Pre-hospital Pathway team: reducing conveyances to the emergency department through patient centered Community Emergency Medicine	Journal Article	Springer	2021	21	Patton, Andrew; O’Donnell, Cathal; Keane, Owen; Henry, Kieran; Cro	1471-227X	10.1186/s12873-021–00536-x	https://doi.org/10.1186/s12873-021-00536-x;
20	The effect of a specialist paramedic primary care rotation on appropriate non-conveyance decisions (SPRAINED) study	Journal Article	The College of Paramedics	2021	7	Pilbery, Richard; Young, Tracey; Hodge, Andrew	Unassigned	10.29045/14784726.2022.06.7.1.9	https://www.ingentaconnect.com/content/tcop/bpj/2022/00000007/00000001/art00003;jsessionid=5glib34gjq4nm.x-ic-live-03
21	The experience of non-conveyance following emergency medical service triage from the perspective of patients and their relatives	Journal Article	ScienceDirect	2021	54	van Doorn, Silvie C. M.; Verhalle, Ruud C.; Ebben, Remco H. A.; Fro	1755-599X	10.1016/j.ienj.2020.100952	https://www.sciencedirect.com/science/article/pii/S1755599X20,301,245;
22	The Safety of Non-Transport Decisions Made by Ambulance Personnel: A Retrospective Study of Subsequent Hospital Admission and 30-Day Mortality	Report	Research Square	2021	1	Amundsen, Kjersti; Elden, Marie Svanes; Myrmel, Lars; Assmus, Jörg;	Unassigned	10.21203/rs.3.rs-276252/v1	https://www.researchsquare.com/article/rs-276252/v1
23	Ambulance crew-initiated non-conveyance in the Helsinki EMS system—A retrospective cohort study	Journal Article	Wiley Online Library	2022	66	Heinonen, Kari; Puolakka, Tuukka; Salmi, Heli; Boyd, James; Laiho,	1399–6576	10.1111/aas.14049	https://onlinelibrary.wiley.com/doi/abs/10.1111/aas.14049;
24	Emergency Medical Services Clinicians’ Perspectives on Pediatric Non-Transport	Journal Article	Taylor and Francis + NEJM	2022	0	Ward, Caleb E.; Singletary, Judith; Hatcliffe, Rachel E.; Colson, Cindy	1090–3127	10.1080/10903127.2022.2108180	https://doi.org/10.1080/10903127.2022.2108180;
25	EMS Non-Transport of Low-Risk COVID-19 Patients	Journal Article	Taylor and Francis + NEJM	2022	0	Couturier, Katherine; Nelson, Alexander R.; Burns, Kevin; Cone, Davi	1090–3127	10.1080/10903127.2022.2083278	https://doi.org/10.1080/10903127.2022.2083278;
26	Trends in fall-related encounters and predictors of non-transport at a US Emergency Medical Services Agency	Journal Article	Wiley Online Library	2022	30	Jeruzal, Jessica N.; Boland, Lori L.; Jin, Diana; Traczyk, Christie L.; S	1365–2524	10.1111/hsc.13613	https://onlinelibrary.wiley.com/doi/abs/10.1111/hsc.13613;
27	Using machine learning to predict subsequent events after EMS non-conveyance decisions	Journal Article	BioMed Central	2022	22	Paulin, Jani; Reunamo, Akseli; Kurola, Jouni; Moen, Hans; Salanterä,	1472–6947	10.1186/s12911-022–01901-x	https://doi.org/10.1186/s12911-022-01901-x;
28	Decision-making in ambulance service non-conveyance – the DMASC survey	Journal Article	Emergency Medicine Journal	2019	36	Sarah Black, Ian Frampton	Unassigned	10.1136/emermed-2019–999.25	https://www.proquest.com/openview/e4fc2aa74cc9181adf7bf5c0b6c0fd44/1?pq-origsite=gscholar&cbl=2,041,072;
29	Convey or not convey? Does crew skill level predict hospital conveyance rate in a UK regional NHS ambulance service trust?	Journal Article	Emergency Medicine Journal	2019	36	Sarah Black, Ian Frampton	Unassigned	10.1136/emermed-2019–999.26	https://www.proquest.com/openview/e4fc2aa74cc9181adf7bf5c0b6c0fd44/1?pq-origsite=gscholar&cbl=2,041,072;

In total, 10 studies were identified from the PubMed database and 72 from Google Scholar. Furthermore, 73.6% (*n* = 53) of studies were excluded from this review. First, 16.6% (*n* = 12)of duplicated articles were excluded by Al-Rayyan® software and 2.7% (*n* = 2) by the reviewers. Second, the reviewers excluded 43% (*n* = 31) of studies with irrelevant backgrounds. Third, 12.5% (*n* = 9) of studies with irrelevant outcomes were removed. Therefore, 36.1% (*n* = 26) of the articles, two theses and one book chapter were ultimately retained as they were considered relevant for this review. The factors related to patient non-conveyance identified after the thorough review of these articles were patient-initiated refusals (PIR), clinical-initiated decisions (CID), and dispatcher-initiated decisions (DID).

PIR refer to situations where the patient, after having initially called for emergency services, refused to be transported to the hospital. These decisions can be due to various reasons, such as perceived improvement in their condition, fear of medical costs, or unwillingness to leave home. Conversely, CID denotes circumstances where the healthcare professionals responding to the emergency call decide not to transport the patient to the hospital. This could occur when the responding clinician assesses the patient’s condition as not requiring further hospital care or deems it more appropriate for the patient to seek alternative care pathways such as primary or community care services. Lastly, DID are instances where the decision for patient non-conveyance is made at the dispatch level. Based on the information provided during the call, this might happen when the dispatcher determines that the situation does not necessitate ambulance transport to the hospital. In such cases, callers might be advised to seek alternative care pathways. Each of these aspects reflects a different point in the emergency services pathway where a decision for non-transport may be made. They underline the multifaceted nature of non-transport decisions in emergency medical services and contribute to our understanding of the complexity of these scenarios.

According to the data presented in Tables 2 and 3, 46.4% (*n* = 51) of the studies included in this review were from North America, 43.6% (*n* = 48) were from Europe, and 7.3% (*n* = 8) were from Australia. The remaining 9% (*n* = 3) were from Asia and Africa.

**Table 2:** Count of non-transport articles included (including the articles cited in the review articles) according to the geographic area

Geographic areas covered	Number of articles
North America	36
Europe	17
USA	13
UK	11
Australia	6
Asia	5
Finland	5
Sweden	4
Netherlands	4
Canada	2
New Zealand	2
Turkey	2
Ireland	2
Africa	1
Australia	1
KSA	1
Norway	1
Total	113

**Table 3:** Geographic areas covered by the included articles’ title

	Title	Geographic areas covered
1.	Can Paramedics Safely Refuse Transport of Non-Urgent Patients?	Turkey = 2, USA = 6, Ireland = 1, UK = 1, Canada = 1
2.	Variation in Interpretation of Guidance as an Explanation of Between-Service Variation in Ambulance Quality Indicators on Non-Conveyance	UK
3.	A patient-safety and professional perspective on non-conveyance in ambulance care: a systematic review	North America (*n* = 36), Europe (*n* = 17), Australia (*n* = 6), Asia (*n* = 5), and Africa (*n* = 1)
4.	Conveyance and non-conveyance to the emergency department after self-harm: Prevalence and ambulance service staff perspectives	Yorkshire UK
5.	Encountering and counselling patients and family members in out-of-hospital emergency care in non-conveyance situations: Follow-up s	Finland
6.	Exploring variation in how ambulance services address non-conveyance: a qualitative interview study	UK
7.	Implementation and use of computerized clinical decision support (CCDS) in emergency pre-hospital care	UK
8.	Non-transport emergency medical service missions – a retrospective study based on medical charts	Finland
9.	Variation in non-conveyance of patients with breathing problems (work package 4.2)	UK
10	Why do ambulance services have different non-transport rates? A national cross-sectional study	UK
11.	Characteristics of non-conveyance ambulance runs: A retrospective study in the Netherlands	Netherlands
12.	Public attitudes towards the preventability of transport and non-transport related injuries: Can a social marketing campaign make a differ	Canada
13.	Patients’ aged ≥ 65 years dispositions during ambulance assignments, including factors associated with non-conveyance to hospital	Sweden
14.	Association Between an Electronic Non-transport Checklist and the Mortality of Patients Discharged-at-Scene by Paramedics in New Ze	New Zealand
15.	EMS non-conveyance: A safe practice to decrease ED crowding or a threat to patient safety?	Finland
16.	Non-Conveyance Due to Patient-Initiated Refusal in Emergency Medical Services: A Retrospective Population-Based Registry Analysis	KSA
17.	Non-conveyance of older adult patients and association with subsequent clinical and adverse events after initial assessment by ambulance	Sweden
18.	Patient experience of non-conveyance following emergency ambulance service response: A scoping review of the literature	USA = 4, Sweden = 2, Australia = 1, New Zealand = 1, Netherlands = 1, Finland = 1, and the United Kingdom = 1
19.	The Alternative Pre-hospital Pathway team: reducing conveyances to the emergency department through patient-centred Community Emergency Medicine	Ireland
20	The effect of a specialist paramedic primary care rotation on appropriate non-conveyance decisions (SPRAINED) study: a controlled int	UK
21.	The experience of non-conveyance following emergency medical service triage from the perspective of patients and their relatives: A qua	Netherlands
22.	The Safety of Non-Transport Decisions Made by Ambulance Personnel: A Retrospective Study of Subsequent Hospital Admission and 3	Norway
23.	Ambulance crew-initiated non-conveyance in the Helsinki EMS system—A retrospective cohort study	Helsinki Finland
24.	Emergency Medical Services Clinicians’ Perspectives on Pediatric Non-Transport	USA
25.	EMS Non-Transport of Low-Risk COVID-19 Patients	USA
26.	Trends in fall-related encounters and predictors of non-transport at a US Emergency Medical Services Agency	USA
27.	Using machine learning to predict subsequent events after EMS non-conveyance decisions	Finland
28.	Decision-making in ambulance service non-conveyance – the DMASC survey	UK
29.	Convey or not convey? Does crew skill level predict hospital conveyance rate in a UK regional NHS ambulance service trust?	UK

## Discussion

Ensuring the provision of effective and safe healthcare within the out-of-hospital environment is an enduring challenge for healthcare professionals. This issue has received considerable attention within North American and European contexts, perhaps attributable to these regions’ established and comprehensive prehospital EMS systems. Moreover, the literature reviewed herein dissected three primary theoretical frameworks that underpin decisions of patient non-transportation: DID, CID, and PIR.

These decision-making mechanisms collectively shape the landscape of patient non-transport decisions in prehospital care. The juxtaposition of these diverse theories underscores the multifaceted and complex nature of non-transport decisions. It hints at the necessity for a nuanced understanding incorporating the varied elements of prehospital care environments.

### Dispatcher-initiated decisions (DID)

Utilizing a standardized computerized system in the triage and management of pre-hospital emergency calls can notably diminish error rates, bolstering quality management and assurance. EMDs often employ a computer-aided dispatch (CAD) system to enhance their decision-making processes, ensuring the expedited dispatch of the most appropriate ERUs. This system undergoes consistent regulation and enhancement, with its performance benchmarks often tethered to the best-performing global EMS systems. A select number of ambulance services employ a computerized medical dispatch system to adeptly manage pre-hospital emergency calls [14, 15], known as the MPDS [16]. MPDS is a computer-based pre-hospital categorization system that can be utilized to optimize the management of pre-hospital cases. It facilitates allocating and dispatching the most appropriate pre-hospital ERU according to the patient’s chief complaints. MPDS enables EMDs to dispatch an ERU staffed with a responder with the required level of skills. This helps avoid delays in providing emergency treatment [17]. A recent study from Finland demonstrated that 40% of emergency calls resulted in patient non-transport decisions; 37.7% of these were aborted by the EMD before the ERU reached the patient [18]. This could be due to causes related to the caller or the EMD.

Additionally, there are instances where the caller decides that EMS assistance is no longer necessary. For example, in some instances, the patient improved or managed their own transportation to the hospital while the EMD still gathered information and processed the case through ProQA. In certain instances, following a comprehensive assessment using ProQA, the EMDs may advise the caller that it is appropriate for the patient to proceed to the nearest healthcare facility using their own means of transportation, if necessary. This is observed in cases where the patient is “not fulfilling” the requirements of an emergency medical condition that mandates immediate pre-hospital medical assistance. Examples of such cases include “asymptomatic hypertension”, “waters were broken for a pregnant woman with no contractions”, or “fever” [19].

MPDS facilitates the EMD going through a detailed medical questionnaire process. This enables them to determine the appropriate protocol and dispatch code according to the information provided by the caller about the patient’s condition. An expert panel continually updates the MPDS using emergency calls from the databases of the best-performing ambulance services worldwide [20]. The determined dispatch code dictates which type of medical or non-medical ERU should be dispatched [20]. A quality improvement study conducted in the USA included cases triaged by the EMDs as not requiring a medical ERU. In such cases, the EMD could dispatch a “non-transport unit.”

Furthermore, based on information provided by the emergency caller and the dispatch code determined by MDPS, the EMD may decide that the dispatch of a highly equipped ERU staffed with advanced healthcare professionals is not required in some instances [21]. Subsequently, they may dispatch a “non-transport unit” as a follow-up unit for patients with minor medical complaints. In a recent study in New York, the researchers demonstrated that the reasons leading to the cancelation of the ERU could be determined based on the information gathered by the EMD during the emergency call process [22]. Occasionally, the caller ends the emergency call without calling back or answering the EMD’s calls [22]. Multiple studies have reported this non-transport by DID [23–28]. Recent systematic reviews have investigated the efficacy of EMS systems utilizing the MPDS and other EMS systems utilizing criteria-based dispatch (CBD). However, published evidence regarding the efficacy of these medical dispatch systems is lacking [1]. Healthcare professionals in some EMS systems under-triaged patients requiring critical care but appropriately identified cases of cardiac arrest [29]. This suggests that not transporting a patient following an emergency call might sometimes be risky.

Several other studies have demonstrated that these systems also under-triage some stroke cases. This is because some of these patients are older adults presenting with non-specific conditions (NSC) which might then be encoded as “sick person” [1]. Other studies have demonstrated that the dispatch code determined by these systems for trauma cases is inconsistent with the patient assessment findings observed by the medical responders [1, 30]. Researchers have also suggested that the anatomical presentation in the dispatch system’s questionnaire would be more effective if appropriately matched with the paramedics’ assessment [30]. In addition, these systems over-triage chest pain, cardiac problems, and complaints of headache [1]. A UK-based study reported that only 5% of priority one dispatch calls with these mentioned complaints were identified as critical [31]. This indicates that EMS resources could be wasted in 95% of non-critical cases that probably did not require conveyance to the emergency department.

### Clinical-initiated decisions (CID)

Clinical determinations enacted by the evaluating medical practitioner may occasionally culminate in a non-transport decision for the patient, a scenario commonly referred to as CID. ‘Non-conveyance’ according to clinician discretion is an outcome that has been cited in many studies (*n* = 15) [18, 23–26, 28, 32–40]. In some instances, non-conveyance represents a clinical verdict enacted by EMS personnel subsequent to their response to an emergency call and the subsequent provision of emergency care to the patient. Consequently, upon the clinical assessment, the responder possesses the discretion to ascertain whether the patient is enduring a non-significant medical condition, obviating the necessity for immediate emergency treatment within a hospital setting. As a result, the patient may not be transported to the hospital.

Notwithstanding, these individuals may be advised to pursue additional medical assistance from an alternate, non-emergency healthcare service or provider. This non-conveyance system has seen widespread adoption among various ambulance services globally [16]. This approach aids in averting unwarranted ambulance conveyances to the hospital for medical conditions that can be effectively addressed in alternate settings, encompassing primary healthcare centers, thereby reducing the undue burden on emergency departments [41]. This system helps avoid emergency department crowding [7, 23, 42, 43].

Nonetheless, the EMS systems adopting this procedure monitor these non-conveyance patients closely by contacting some of them later for follow-up. Non-conveyance rates are also used as a quality indicator within these systems [23, 42, 43]. Furthermore, studies have demonstrated that serious cases might sometimes be miss-triaged as non-conveyance, specifically in older adult patients [44]. A recent study from Sweden demonstrated that NSC is mainly related to older patients. These patients were generally present with stable vital signs. Also, they reported complaints of “affected general health condition,” “general malaise,” “sense of illness,” or “just being unable to cope with daily activities” without providing a more specific chief complaint [44].

Consequently, these patients might be triaged as not requiring critical care. However, they might experience serious health outcomes without immediate treatment and care. Previous studies in the EMS setting have reported that at least one in three NSC patients presented with a serious health issue requiring close hospital monitoring [45, 46].

### Patient-initiated refusal (PIR)

The non-transport decision can also stem from PIR [18, 27, 36, 47–57]. Contemporary studies have illuminated instances where, within numerous EMS systems, patients elect not to be conveyed to the hospital, against clinical advice [56]. In specific EMS systems, for example, in the USA, pre-hospital healthcare workers can acknowledge the PIR only after an online consultation with the medical management team [33, 58]. These PIRs are frequently correlated with a patient’s incapacity to shoulder prospective transport fees, especially in specific jurisdictions where the individual bears such costs. Additional deterrents encompass protracted wait times encountered within the emergency department. In some instances, PIRs are precipitated by patient contentment with the caliber of pre-hospital medical assistance they receive, juxtaposed against their dissatisfaction with the drawn-out procedural rigmarole anticipated at the emergency department [7, 56, 59, 60]. In a recent Middle-East study conducted by the National Ambulance Service of Riyadh, 35.5% of the pre-hospital emergency calls ended with PIR, compared with only 8.8% of patient non-conveyance due to CID [56].

Psychological considerations also significantly come into play, particularly with elderly patients who might harbor apprehension towards polypharmacy. As a result, physicians encounter challenges when prescribing a higher quantity of medications, sometimes as much as 25 pills, for older adults and persuading them to return home when everything seems to be in order [61]. Furthermore, a Swedish study demonstrated that with the increase in the age of patients visiting emergency departments for emergency care, hospitalization, and mortality rates also increase. This is because, in some instances, older patients only visit the emergency department when their medical condition becomes critical [62]. Therefore, many health systems worldwide have recognized the impact of patient non-transport, both for the health outcomes of older adult patients and as a quality indicator in EMS systems. They also reflect a significant challenge that could compromise patients’ health conditions in major and minor trauma cases [63]. Some concerns about the non-conveyance of older patients have been expressed since they can be easily under-triaged. Many older patients not transported to the hospitals, called the emergency services again, and were eventually transported and admitted to the hospital [45, 46, 64]. Some recent studies have focused on the non-conveyance of elderly trauma victims. With their vulnerable physio-pathological conditions, older patients can present with significant trauma even after incidents with low-impact mechanisms. Older patients might also be often misdiagnosed [46].

Meta-analyses have demonstrated that patient non-conveyance mainly affects younger than older patients [64, 65]. Furthermore, these studies have indicated that more than a quarter of the non-transported patients accessed alternative healthcare service providers other than those working in emergency departments (e.g., private clinics) [64].

### Synthesis of recommendations from analyzed studies

Inferences drawn from a comprehensive examination of prior studies reveal that specific EMS systems have integrated the concept of patient non-conveyance into their guidelines. They deem it a practice with an acceptable level of risk, contingent on initiating a telephonic medical consultation or deploying follow-up units for non-conveyance cases [32, 38, 49, 66]. Notably, the term ‘acceptable’ risk elicits diverse interpretations across the literature [37, 38, 50, 67]. There is a latent risk of under-triage, potentially leading to overlooked life-threatening complaints. This is especially pertinent for elderly patients, who might necessitate urgent medical attention within a brief interval [39, 50].

As such, the predominant perception within EMS systems classifies patient non-conveyance to a hospital as an adverse event that could compromise their health outcomes [25, 37–39, 48, 51, 53, 65, 68].

In light of these observations, we concur with the call for precautionary patient transportation to hospitals or implementing a reliable medical follow-up mechanism. It is crucial to clarify that this conclusion hinges on our interpretation of the reviewed literature and advocates for further empirical exploration.

### Limitations

Our study recognizes and acknowledges its intrinsic limitations. Primarily, our investigative approach is a narrative review instead of a systematic exploration of the extant literature. This method, though enabling an encompassing overview of the subject matter, is potentially susceptible to selection bias during the process of literature analysis, which may engender considerable distortions in our resultant findings. Second, the non-conveyance decisions reported in the literature could be affected by factors such as the worldwide diversity of the EMS operational systems. This could also affect the proportion of non-conveyance decisions. Further, generalizing the non-conveyance theories could be difficult as it is also affected by many social, ethnic, and cultural factors and the diversification of worldwide EMS systems. The widespread diversity in policy and practice inherently constrains the universal applicability of our observations and recommendations. As such, we advocate for future research to engage in a more systematic review methodology. Such an approach could help address these potential biases and facilitate a more thorough comprehension.

## Conclusions

Over the past decade, patient non-conveyance to hospitals has surfaced as a significant healthcare concern within pre-hospital environments. While certain studies advocate non-conveyance as a safe practice, others underscore its potential implications on patient safety, potentially compromising healthcare outcomes. In addition to patient safety, non-conveyance can impact the efficiency of the health system by expending resources on potentially unnecessary dispatches of pre-hospital response units. We underscore the need for further research to understand this issue and define its variables comprehensively. Utilizing advanced research methodologies, such as machine learning, can prove instrumental in this exploration. Doing so could enhance clinical decision-making processes and optimize resource utilization, thereby striving to improve both patient outcomes and system efficiency.

## Data Availability

The datasets generated during and/or analyzed during the current study are available from the corresponding author upon reasonable request.

## Abbreviations

EMS: Emergency Medical Services
EMD: Emergency Medical Dispatcher
ProQA: Program Question Answer
ERU: Emergency Response Unit
MPDS: Medical Priority Dispatch Systems
PIR: Patient-initiated refusals
CID: Clinical-Initiated Decisions
DID: Dispatcher-Initiated Decisions
